# H_2_O_2_-preconditioned human adipose-derived stem cells (HC016) increase their resistance to oxidative stress by overexpressing Nrf2 and bioenergetic adaptation

**DOI:** 10.1186/s13287-020-01851-z

**Published:** 2020-08-03

**Authors:** Patricia Garrido-Pascual, Ana Alonso-Varona, Begoña Castro, María Burón, Teodoro Palomares

**Affiliations:** 1 Department of Cell Biology and Histology, Faculty of Medicine and Nursing, University of the Basque Country (UPV/EHU), Leioa, Bizkaia Spain; 2 Histocell, Bizkaia Science and Technology Park, Derio, Bizkaia Spain; 3 Department of Surgery, Radiology and Physical Medicine, Faculty of Medicine and Nursing, University of the Basque Country (UPV/EHU), Leioa, Bizkaia Spain

**Keywords:** Human adipose-derived stem cells, H_2_O_2_ preconditioning, Oxidative stress, Nrf2, Bioenergetic, Cell therapy

## Abstract

**Background:**

Mesenchymal stem cells, including those derived from human adipose tissue (hASCs), are currently being widely investigated for cell therapy. However, when transplanted at the site of injury, the survival and engraftment rates of hASCs are low, mainly due to the harsh microenvironment they encounter, characterized by inflammation and oxidative stress. To overcome these therapeutic limitations, cell preconditioning with low-concentration of hydrogen peroxide (H_2_O_2_) has been proposed as a plausible strategy to increase their survival and adaptation to oxidative stress. Nonetheless, the underlying mechanisms of this approach are not yet fully understood. In this study, we analyzed molecular and bioenergetic changes that take place in H_2_O_2_ preconditioned hASCs.

**Methods:**

Long-term exposure to a low concentration of H_2_O_2_ was applied to obtain preconditioned hASCs (named HC016), and then, their response to oxidative stress was analyzed. The effect of preconditioning on the expression of Nrf2 and its downstream antioxidant enzymes (HO-1, SOD-1, GPx-1, and CAT), and of NF-κB and its related inflammatory proteins (COX-2 and IL-1β), were examined by Western blot. Finally, the Seahorse XF96 Flux analysis system was used to evaluate the mitochondrial respiration and glycolytic function, along with the total ATP production.

**Results:**

We found that under oxidative conditions, HC016 cells increased the survival by (i) decreasing intracellular ROS levels through the overexpression of the transcription factor Nrf2 and its related antioxidant enzymes HO-1, SOD-1, GPx-1, and CAT; (ii) reducing the secretion of pro-inflammatory molecules COX-2 and IL-1β through the attenuation of the expression of NF-κB; and (iii) increasing the total ATP production rate through the adaption of their metabolism to meet the energetic demand required to survive.

**Conclusions:**

H_2_O_2_ preconditioning enhances hASC survival under oxidative stress conditions by stimulating their antioxidant response and bioenergetic adaptation. Therefore, this preconditioning strategy might be considered an excellent tool for strengthening the resistance of hASCs to harmful oxidative stress.

## Background

Over recent decades, mesenchymal stem cells (MSCs) have been widely used in cell therapy because of their immunomodulatory and anti-inflammatory properties and their well-documented cytoprotective and reparative effects [1–3]. Unfortunately, however, the rates of survival and engraftment of MSCs are low, mainly due to harsh environmental conditions they encounter on implantation such as nutrient deprivation, inflammation, and oxidative stress [4, 5].

In particular, oxidative stress, caused by antioxidant depletion or/and reactive oxygen species (ROS) accumulation [6], leads to cell damage and dysfunction, which decrease the viability and immunomodulatory activity of engrafted MSCs [7, 8]. Cells have developed several strategies to cope with oxidative stress, most involving transcription factors that promote the expression of antioxidant response elements. Nonetheless, under pathological conditions, antioxidant systems may be overwhelmed [6].

A major cellular mechanism to reduce oxidative stress is via the nuclear factor E2-related factor 2 (Nrf2)-antioxidant response element (ARE) signaling pathway. Nrf2 is a transcription factor that regulates the expression of genes coding for antioxidant, anti-inflammatory, and detoxifying proteins [9, 10]. In the absence of stress conditions, Nrf2 localizes in the cytoplasm where it interacts with the actin-binding protein, Kelch-like ECH-associated protein 1 (Keap1), leading to its ubiquitination and proteasomal degradation [11]. On the other hand, when signals from ROS target the Nrf2-Keap1 complex, Nrf2 dissociates from Keap1 and translocates into the nucleus inducing transcription of a wide range of proteins, such as heme oxygenase-1 (HO-1), superoxide dismutase-1 (SOD-1), glutathione peroxidase-1 (GPx1), and catalase (CAT), that play an important role in protecting cells against oxidative stress-induced damage [12–14]. In addition to its involvement in antioxidant and detoxifying responses, Nrf2 also plays an important role in inflammation. Recent studies have shown crosstalk between Nrf2 and nuclear factor-κB (NF-κB) signaling pathways under stress [15]. NF-κB is an inflammatory transcription factor that, when translocated to the nucleus, initiates the transcription of proinflammatory molecules including cytokines (IL-1, IL-6, TNF-α), cyclooxygenase-2 (COX-2), and others [16]. It can be activated by oxidative stress or inhibited by the presence of antioxidant agents. Specifically, the activation of Nrf2 prevents the overproduction of proinflammatory mediators, whereas its inhibition is associated with enhanced expression of NF-ĸB [17–19]. Overall, the aforementioned findings indicate that the interaction between these two pathways is closely related to the oxidative/inflammatory state of the cell and thus to its survival.

Survival in an oxidative/inflammatory environment is an energy-demanding process for MSCs that requires effective metabolic adaptation to fulfill the bioenergetic demand [20, 21]. Bioenergetics plays a central role in tolerance to environmental stress, a balance between the input and expenditure of energy being a key requirement for survival [22]. Cells obtain free energy in chemical form through the catabolism of nutrient molecules and use this energy to produce ATP from ADP and Pi. The hydrolysis of ATP releases free energy that cells use to maintain functions such as synthesis of proteins from amino acids and nucleic acids from nucleotides, transport of molecules or ions against a gradient across membranes, and cell motility and division. In mammalian cells, glycolysis and mitochondrial oxidative phosphorylation (OXPHOS) pathways provide most cellular ATP. The majority of cells can readily switch between these two pathways, thereby adapting to changes in their microenvironment [23]. In order to survive, MSCs must adapt their metabolism to maintain bioenergetic efficiency under unfavorable conditions.

As a means to strengthen MSC adaptability in an oxidative/inflammatory environment, thereby increasing the survival rate after implantation, various cell preconditioning strategies have been tested [24–27]. The preconditioning process—sub-lethal exposure to cellular stressors—promotes the expression and secretion of certain molecules that are required to reduce damage and increase survival, giving cells the capacity to respond efficiently to a higher level of the same stressor [28–30]. In addition, a previous study by our group showed that preconditioning with low doses of H_2_O_2_ promoted faster recovery after cryopreservation of human adipose-derived mesenchymal stem cells (hASCs) enhancing their adhesion, migration, and survival under oxidative stress [31].

Given these previous findings, the present in vitro study sought to elucidate the bioenergetic and molecular mechanisms underlying the adaptation and survival of H_2_O_2_-preconditioned hASCs (called HC016) in an oxidative stress-induced model.

## Materials and methods

### Cell culture

Human adipose-derived stem cells (hASCs) were kindly donated by Histocell S.L. (Science and Technology Park of Bizkaia, Spain). These cells were maintained in DMEM Glutamax™ (Dulbecco’s modified Eagle medium, Gibco, Paisley, UK) supplemented with gentamicin (1 μl/ml, Sigma-Aldrich, St. Louis, MO, USA) and 10% heat-inactivated fetal bovine serum (FBS, Biochrom, Berlin, Germany) and incubated at 37 °C, in a humidified atmosphere containing 5% CO_2_. Cells up to passage 4 were used in this study.

### H_2_O_2_ preconditioning of hASCs

Long-term exposure to a low concentration of H_2_O_2_ (PanReac AppliChem, Barcelona, Spain) was applied to obtain H_2_O_2_-preconditioned hASCs (HC016 cells). Briefly, hASCs were exposed to 10 μM of H_2_O_2_ for 7 days, with replenishment of oxidative culture media twice during the preconditioning protocol (see details in HC016 patent; WO/2013/004859, 2013). Non-preconditioned hASCs were cultured in parallel for the same number of passages. Once the preconditioning process had been completed, HC016 cells and hASCs were seeded at high density and incubated at 37 °C in a humidified atmosphere containing 5% CO_2_ for 18–20 h in complete medium, until use in experiments.

### Oxidative stress induction

Oxidative stress was induced by administering a moderate (0.25 mM) or high (0.5 mM) dose of H_2_O_2_. For all the experiments, HC016 cells and hASCs were exposed to H_2_O_2_ (0.25 or 0.5 mM) in DMEM Glutamax™ without FBS, at 37 °C in a humidified atmosphere containing 5% CO_2_ for 1 h. After that, the media were replaced with fresh FBS-free DMEM-Glutamax™.

### Detection of intracellular ROS, apoptosis analysis, and cytotoxicity assay

To determine whether HC016 cells were more resistant to oxidative stress than hASCs, cells were exposed to a moderate or high dose of H_2_O_2_ for 1 h, and values of ROS, cytotoxicity, and apoptosis were assessed.

#### Measurement of ROS

Intracellular levels of ROS in HC016 cells and hASCs were detected using 2′-7′-dichlorofluorescein diacetate (H2-DCF-DA, Molecular Probes, Eugene OR, USA). As a probe, H2-DCF-DA was added to the cells at a final concentration of 10 μM, at 37 °C for 30 min. After this period, the fluorescent probe was removed and cells were washed with 1× PBS. Finally, for 1 h, HC016 cells and hASCs were exposed to different H_2_O_2_ concentrations (0.25 or 0.5 mM) and intracellular ROS accumulation was measured every 10 min in a microplate reader (λexcitation [λex] = 492–495; λemission [λe] = 517–527 nm). The results obtained were normalized to the number of cells and expressed as the mean ± SD of at least three independent experiments performed in quintuplicate.

#### Apoptosis analysis

Apoptosis was analyzed by flow cytometry using an Alexa Fluor 488 Annexin V/PI Dead Cell Apoptosis Kit (Thermo Fisher Scientific, Eugene, OR, USA). HC016 cells and hASCs were harvested 24 h after the 1-h moderate and high H_2_O_2_ exposure and stained with Alexa Fluor 488 Annexin and PI for 15 min at room temperature (RT) (1 × 106 cells/mL). Then, stained cells were analyzed by flow cytometry, measuring the fluorescence (λex = 488; λem = 530 and 575 nm, respectively). Data were analyzed using Flowing Software (Turku Centre for Biotechnology, University of Turku, Finland) and are reported as the mean ± SD of three independent experiments. Histograms are representative of these experiments.

#### Cytotoxicity assay

Cytotoxicity was determined by the quantification of extracellular LDH. For this assay, 4 × 103 cells were seeded in a 96-well plate. LDH assays were performed according to the manufacturer’s protocol (Cytotoxicity Detection Kit, Roche, Mannheim, Germany). Briefly, 24 and 48 h after the 1-h moderate or high oxidant exposure, cytotoxicity was assessed by measuring LDH release from damaged cells into culture media. The kit reaction mixture was added to each well (1:2 dilution), and the plate was incubated in darkness at RT for 30 min. Finally, the absorbance was measured in a microplate reader at *λ* = 490 nm. Data were normalized to cells cultured with 1% v/v Triton X-100 (100% death), and cytotoxicity was calculated as a percentage of the untreated control cells. Assays were performed at least three times (*n* ≥ 4).

### Western blot analysis

HC016 cells and hASCs were collected at 0 or 24 h after the 1-h H_2_O_2_ insult. Then, cells were lysed in 1x Laemmli buffer (Sigma-Aldrich, St. Louis, MO, USA) and sonicated to obtain a homogeneous sample. Whole-cell preparations and nuclear extracts were prepared employing an adaptation of a high-quality biochemical fractionation protocol described elsewhere [32]. Briefly, cells were pelleted and resuspended in cytoplasmic extraction buffer (20 mM Tris, pH 7.6, 0.1 mM EDTA, 2 mM MgCl2·6H2O, 0.5 mM Na3VO4) to induce hypotonic swelling. To release cytoplasmic proteins, Nonidet P-40 (Igepal, Sigma-Aldrich, St. Louis, MO, USA) was added to a final concentration of 1%. The cytoplasmic extract was separated by centrifugation (500*g* for 3 min, at 4 °C), and the pellet containing nuclei was resuspended in 1% Nonidet P-40 cytoplasmic extraction buffer and centrifuged at 4 °C and 500*g* for 3 min; this washing step was repeated once more to obtain a pellet of pure nuclei. Protein quantification was performed by trichloroacetic acid (TCA) precipitation (Fluka Biochemika, Steinheim, Germany).

Protein lysates were boiled for 5 min, separated on 10% SDS-PAGE and transferred onto a nitrocellulose membrane (GE Healthcare, Life Sciences, Freiburg, Germany). Membranes were blocked with 5% skimmed milk in TBST (20 mM Tris, 500 mM NaCl, 0.1% Tween-20 (v/v), pH 7.5) for 1 h and, subsequently, incubated overnight at 4 °C with primary antibodies against Nrf2 (1:1000), SOD-1 (1:1000), HO-1 (1:1000), GPx1 (1:1000), CAT (1:1000), NF-κB (1:1000), Lamin A/C (1:5000, Genetex, Irvine, CA, USA), COX-2 (1:1000, Abcam, Cambridge, UK), IL-1β (1:1000, R&D Systems, Inc., Minneapolis, MN, USA), HIF-1α (1:250, BD Biosciences, San Jose, CA, USA), and β-Actin (1:5000, EMD Millipore, Darmstadt, Germany). After washing, membranes were incubated with the corresponding secondary antibody, goat anti-rabbit IgG, rabbit anti-mouse IgG (1:1000, Thermo Fisher Scientific, Waltham, MA, USA), or donkey anti-goat IgG (1:1000, Bethyl Laboratories, Montgomery, TX, USA) for 1 h at RT. Finally, membranes were visualized using SuperSignal West Pico PLUS Chemiluminescent Substrate (Thermo Fisher Scientific, Waltham, MA, USA). Images were acquired with the G:Box Chemi HR16 gel documentation system (Syngene, Frederick, MD, USA), and densitometry was performed with ImageJ (NIH, Bethesda, MD, USA). Densitometry values were then normalized to that of the corresponding loading controls. HC016 cell data were expressed relative to hASCs and are reported as the mean ± SD of at least three different experiments.

### Assessment of mitochondrial stress

MitoTracker®Red CMXRos (Invitrogen, Eugene, OR, USA), a derivative of X-rosamine, was used as a probe to assess mitochondrial stress. This probe labels mitochondria depending on the mitochondrial membrane potential (MMP) and gives information on mitochondria morphology and stress. For this experiment, cells were seeded in 96-well plates or μ-Slides with 8 wells (Ibidi GmbH, Martinsried, Germany); 24 h after the H_2_O_2_ exposure period, they were incubated with 100 mM MitoTracker® probe for 30 min at 37 °C. For mitochondria visualization, samples were examined under a Zeiss LSM880 Airyscan confocal microscope (Carl Zeiss Inc., Chicago, IL, USA) using a × 40 objective. For MMP quantification, the fluorescence intensity of living cells was measured in a microplate reader (λex = 579; λem = 599 nm). The results obtained were normalized to the number of cells and are given as the mean ± SD of at least three independent assays (*n* ≥ 3).

### Cellular bioenergetic measurements

To explore whether preconditioning altered the bioenergetic profile of hASCs, Seahorse XF Cell Mito Stress Tests, XF Glycolytic Rate Assays, and XF Real-Time ATP Rate Assays were performed using a Seahorse XFe96 Extracellular Flux Analyzer (Agilent Technologies, Santa Clara, CA, USA) following the manufacturer’s instructions. HC016 cells and hASCs were plated on an XF96 cell culture microplate, and 24 h after the oxidative insult, cells were tested for both oxygen consumption rate (OCR) and extracellular acidification rate (ECAR) in XF DMEM Base Medium without phenol red, supplemented with 10 mM glucose, 2 mM glutamine, 1 mM pyruvate, and 5 mM HEPES, pH 7.4. For each assay, OCR and ECAR were measured before (basal conditions) and after sequential administration of different metabolic stressors. Specifically, measurements of mitochondrial function (basal respiration, maximal respiration, ATP-linked respiration, and coupling efficiency), the XF Cell Mito Stress Tests, were used (1.5 μM oligomycin, an ATP synthase inhibitor; 0.5 μM FCCP, an uncoupling agent that collapses the proton gradient and disrupts the mitochondrial membrane potential; and 0.5 μM of rotenone and antimycin A; ROT/AA). Further, to measure key parameters of glycolytic rate for basal conditions and compensatory glycolysis following mitochondrial inhibition were acquired using the XF Glycolytic Rate Assays (0.5 μM ROT/AA, a respiratory chain inhibitor, and 50 mM of 2-deoxy-D-glucose; 2-DG, glycolytic inhibitor). Finally, to determine the total cellular ATP production rate, as well as the fractional contributions from glycolysis and mitochondrial respiration, we used the XF Real-Time ATP Rate Assays (1.5 μM oligomycin and 0.5 μM of ROT/AA). The results obtained were normalized to the number of cells and analyzed by Wave Desktop Software 2.6 (Agilent Technologies, Cedar Creek, TX, USA). All assays were performed at least three times (*n* ≥ 3), and results are presented as means ± SD.

### Statistical analysis

The number of samples analyzed is reported for each experiment. All data are presented as mean ± SD. Statistical analysis was performed using GraphPad Prism statistical software (version 5.0; GraphPad Software). Significance was assessed using analysis of variance followed by Bonferroni’s post hoc test and *t* tests, as appropriate. Statistical differences were considered significant where *p* < 0.05. All the figures presented here represent the data obtained in at least three independent experiments with similar results.

## Results

### Preconditioning protects cells against oxidative stress

**Fig. 1:**
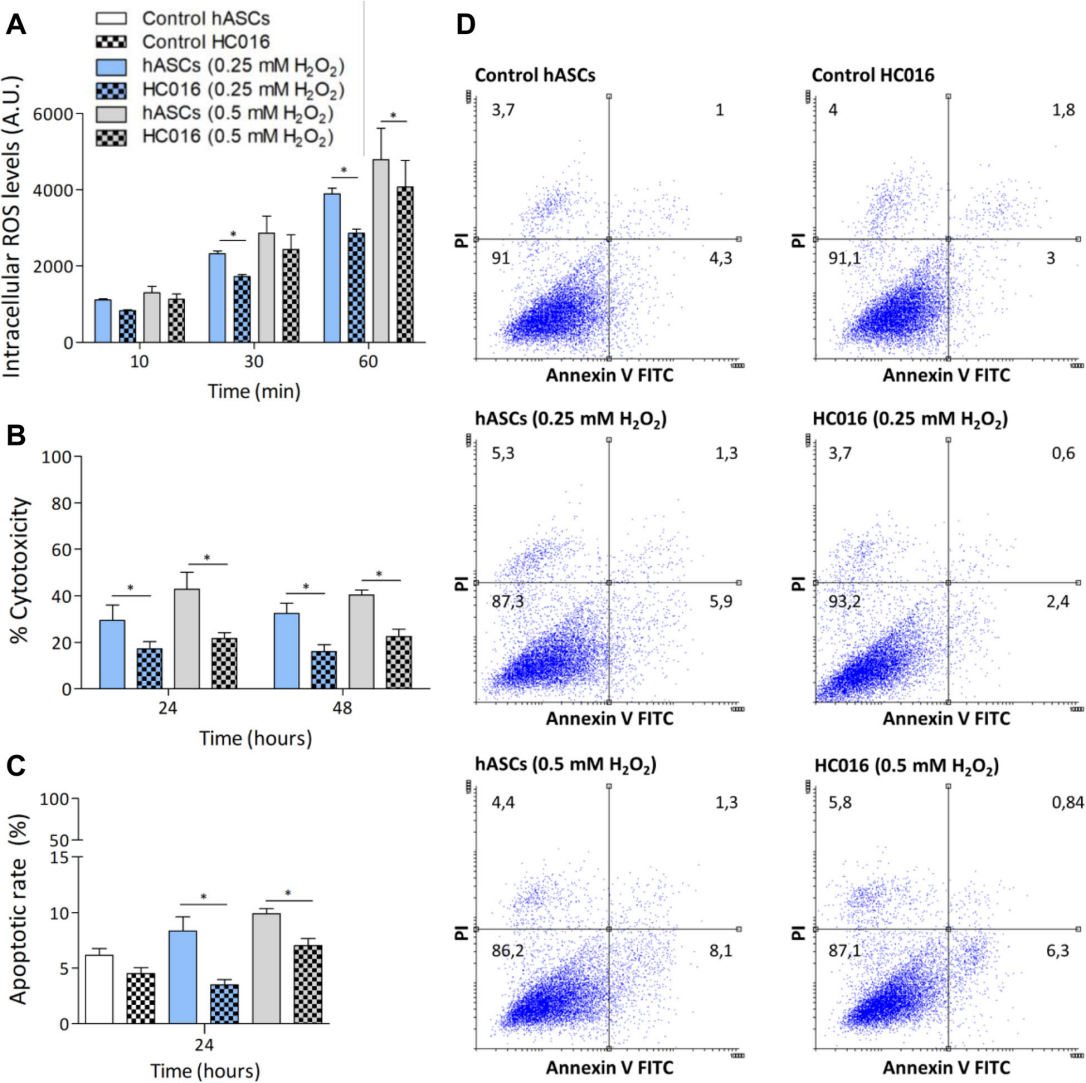
Cytoprotective effect of preconditioning in an oxidative stress-induced model (0.25 or 0.5 mM H_2_O_2_). HC016 cells had higher cell survival capacity than hASCs. **a** ROS generation was induced by H_2_O_2_ and measured every 10 min for 1 h. Histogram shows that ROS levels were attenuated in HC016 cells. **b** Measurements of lactate dehydrogenase (LDH) release at 24- and 48-h post-stimulus showed that HC016 cells exhibited a significant reduction in LDH release at all post-stimulus times analyzed. **c** Quantitation of cells undergoing early and late apoptosis. **d** Representative graphs of annexin V/PI assay performed by flow cytometry. Annexin V−/PI− represents live cells, annexin V+/PI− early apoptosis, annexin V+/PI+ late apoptosis, and annexin V−/PI+ necrosis. Experiments were performed at least in triplicate in three independent experiments. Data are expressed as mean ± SD. *p < 0.05

To evaluate the cytoprotective effect of the H_2_O_2_ preconditioning, cells were incubated with 0.25 or 0.5 mM H_2_O_2_ without FBS for 1 h. During this period, we evaluated ROS levels and observed that, although the levels increased in a time- and dose-dependent manner in both HC016 cells and hASCs, they were significantly lower in the case of HC016 cells at 30 and 60 min when exposed to 0.25 mM H_2_O_2_ and at 60 min when exposed to 0.5 mM H_2_O_2_ (Fig. 1a). In addition, after 24 and 48 h, preconditioning was associated with significantly lower H_2_O_2_-induced cytotoxicity in HC016 cells. Specifically, at 24 h, HC016 cells exposed to 0.25 or 0.5 mM H_2_O_2_ showed, respectively, a 1.7- and 1.9-fold lower cytotoxicity percentage than hASCs. At 48 h, the reduction in LDH release was maintained when compared to that in hASCs, the cytotoxicity percentage being 2- and 1.8-fold lower in HC016 cells exposed to 0.25 or 0.5 mM H_2_O_2_, respectively (Fig. 1b). Moreover, as shown in Fig. 1c, H_2_O_2_-exposed HC016 cultures contained a significantly lower percentage of apoptotic cells than the oxidized hASC cultures. In particular, a 2.4-fold reduction was achieved in the case of cultures exposed to 0.25 mM H_2_O_2_ (HC016 cells 3.5 ± 0.4% and hASCs 8.3 ± 1.2%) and 1.4-fold reduction in those exposed to 0.5 mM H_2_O_2_ (HC016 cells 7 ± 0.6% and hASCs 9.9 ± 0.4%; Fig. 1d).

### Preconditioning promotes antioxidant response and reduces pro-inflammatory protein expression

**Fig. 2:**
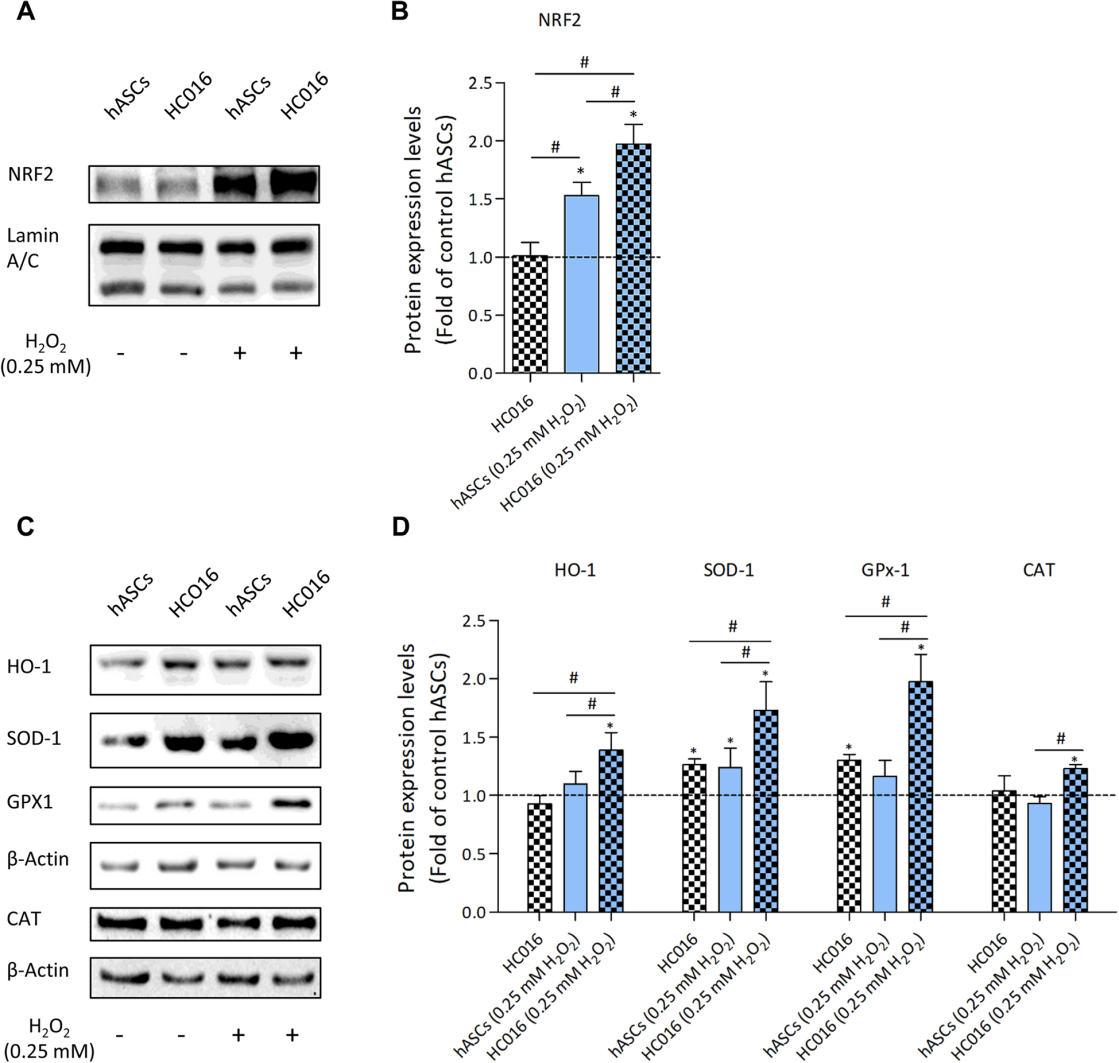
Preconditioning enhanced antioxidant response. HC016 cells and hASCs were exposed to 0.25 mM H_2_O_2_ for 1 h in FBS-free media and lysed just after the insult. **a** Expression and **b** quantification of nuclear Nrf2. **c** Expression and **d** quantification of HO-1, SOD-1, GPx1, and CAT. Values were normalized to their corresponding loading control. At least three different independent experiments were performed. Data were normalized to control hASCs (dotted line) and expressed as mean ± SD. *p < 0.05, compared with control hASCs, ^#^*p* < 0.05

To analyze the antioxidant effect of preconditioning, the expression of Nrf2 and several anti-oxidant enzymes (HO-1, SOD-1, GPx-1, and CAT) was studied by Western blot immediately after the 1-h H_2_O_2_ insult. The results revealed that after exposure to 0.25 mM H_2_O_2_, nuclear Nrf2 expression increased (Fig. 2a), the magnitude of this increase being 1.3-fold larger in HC016 cells than hASCs (Fig. 2b). Notably, preconditioned cells also exhibited an enhancement in antioxidant enzyme expression (Fig. 2c). In contrast with hASCs, HC016 cells exposed to 0.25 mM H_2_O_2_ showed a 1.3-fold increase in HO-1 and CAT expression, 1.4-fold increase in SOD-1 expression, and 1.7-fold increase in GPx-1 expression (Fig. 2d).

**Fig. 3:**
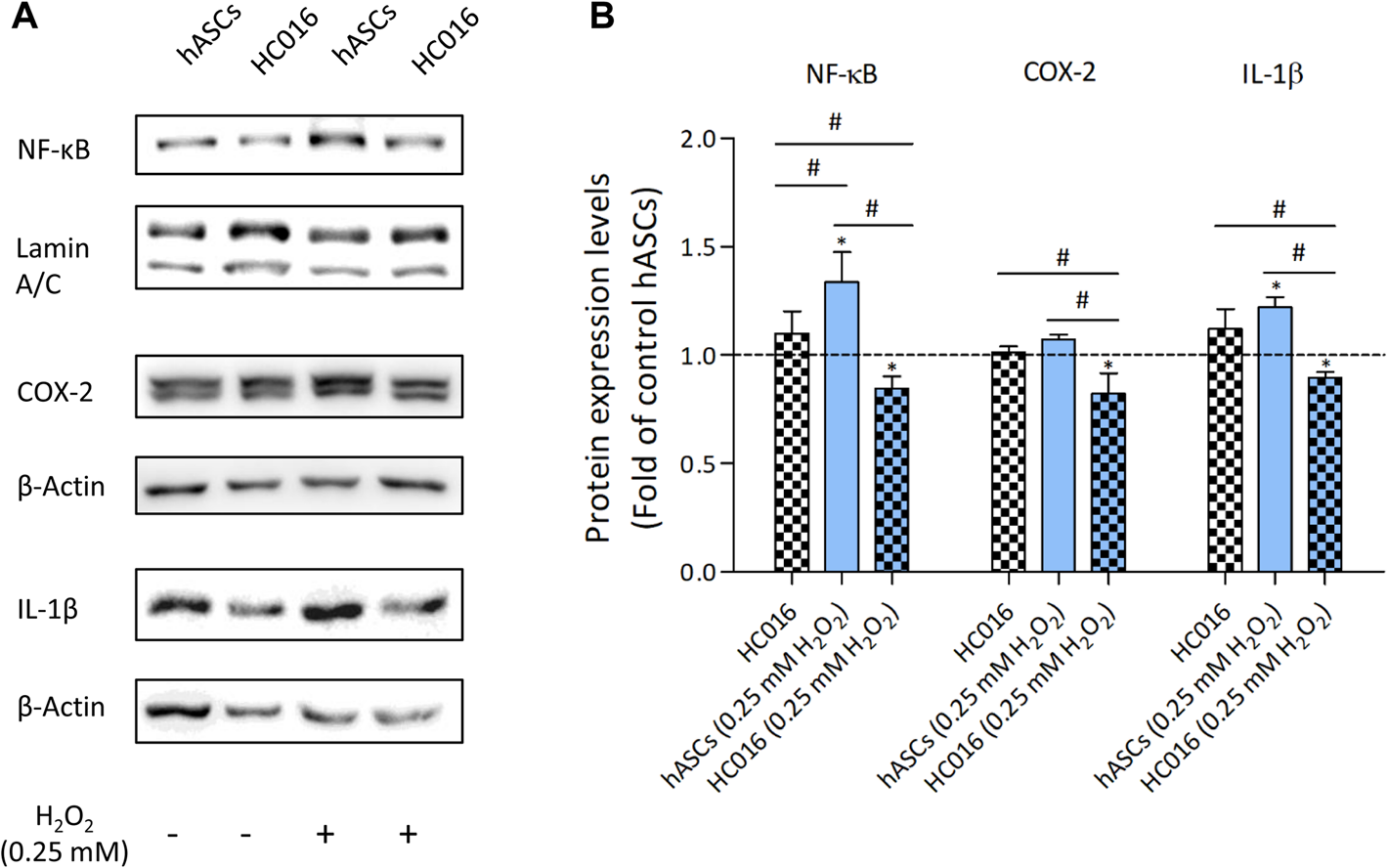
Cell preconditioning reduces the expression of proinflammatory proteins. **a** Expression of NF-κB, COX-2, and IL-1β. HC016 cells and hASCs were exposed to 0.25 mM H_2_O_2_ for 1 h and lysed 24 h after the insult. **b** Expression and quantification of NF-κB, COX-2, and IL-1β. Values were normalized to β-actin. At least three different experiments were performed. Data were normalized to control hASCs (dotted line) and expressed as mean ± SD. *p < 0.05, compared with control hASCs, ^#^*p* < 0.05

To evaluate how preconditioning affected inflammatory protein expression, we measured the expression of NF-κB and proinflammatory molecules COX-2 and IL-1β 24 h after the oxidative insult by Western blot. The results showed that preconditioning attenuated the expression of all these proteins when cells were exposed to oxidative stress (Fig. 3a). As shown in Fig. 3b, whereas no differences were observed between control groups, HC016 cells exhibited a significantly lower expression of NF-κB, COX-2, and IL-1β than hASCs (1.6, 1.3, and 1.4-fold lower, respectively).

### Preconditioning modulates cell metabolism

**Fig. 4:**
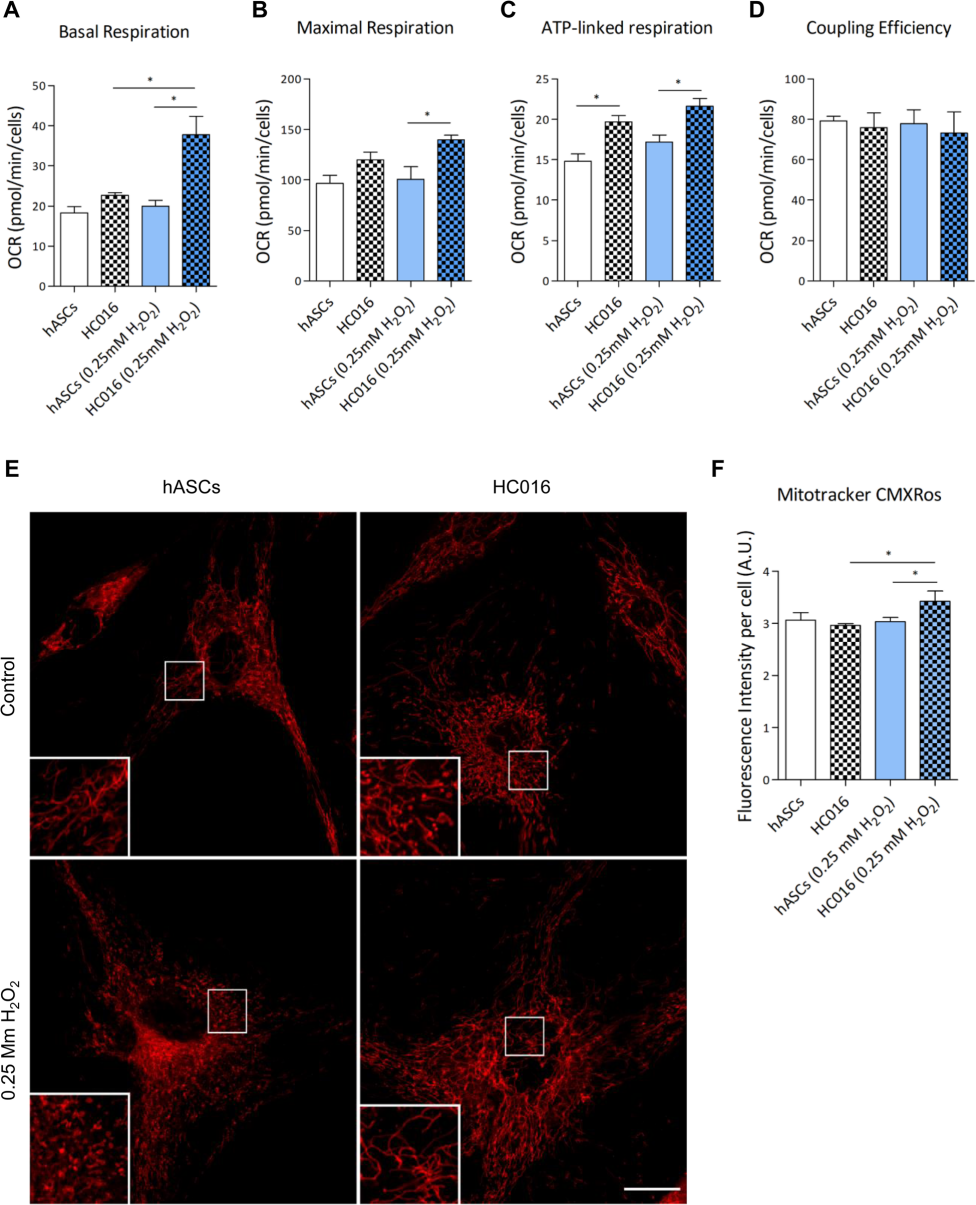
Mitochondrial respiration parameters. Seahorse XF Cell Mito Stress Test was performed in HC016 cells and hASCs under control conditions or after 0.25 mM H_2_O_2_ exposure. **a** Basal respiration. **b** Maximal respiration. **c** ATP-linked respiration. **d** Coupling efficiency. Mitochondrial morphology and MMP in HC016 cells and hASCs. **e** Confocal microscopy images of HC016 cells and hASCs labeled live with MitoTracker Red CMXRos (red, mitochondria). **f** MMP was quantified in a plate reader and normalized to cell number. At least three different independent experiments were performed, and results were expressed as mean ± SD. *p < 0.05

We investigated whether preconditioning had any impact on OXPHOS or glycolytic metabolism of hASCs under standard conditions and in response to oxidative stress. To examine mitochondrial function, the XF Cell Mito Stress Test was used. After 0.25 mM H_2_O_2_ exposure and compared to hASCs, preconditioned cells displayed significantly higher basal mitochondrial oxygen consumption (1.8-fold higher; Fig. 4a) and maximal respiratory capacity (1.4-fold higher; Fig. 4b). Moreover, when exposing cells to oligomycin, an inhibitor of ATP synthase, we also detected an increase in ATP-linked respiration for HC016 cells (1.3-fold) and HC016 cells exposed to 0.25 mM H_2_O_2_ (1.25-fold), compared with non-preconditioned cells (Fig. 4c), indicating that preconditioned cells have an enhanced energy capacity to respond to stress. Regarding coupling efficiency, both cell types exhibited an efficiency of more than 70% in basal and in oxidative conditions and there were no significant differences between them (Fig. 4d).

In addition, to assess mitochondrial stress, mitochondrial morphology and MMP were analyzed with MitoTracker®Red CMXRos probe. In Fig. 4e, we observed that mitochondria of control hASCs and HC016 cells presented a similar elongated and tubular shape. When cells were exposed to 0.25 mM H_2_O_2_, the morphology of hASC and HC016 cell mitochondria differed. HC016 cell mitochondria maintained the same tubular shape as the controls, whereas hASC mitochondria looked fragmented (Fig. 4e). Further, considering microscopy images, HC016 cells seemed to increase their mitochondrial mass, and the maintenance of their tubular shape after the oxidative insults reveals a strongly interconnected network distributed uniformly through the cytoplasm, suggesting a strengthening of fusion processes, probably to enhance ATP production. Regarding MMP, no significant differences were observed between control hASCs and HC016 cells; however, when the cells were exposed to a moderate oxidative insult, HC016 cells exhibited a higher MMP than their corresponding control (16 ± 1.2% higher) and oxidized non-preconditioned cells (14 ± 1.2% increase), these findings correlating with the confocal images (Fig. 4f).

**Fig. 5:**
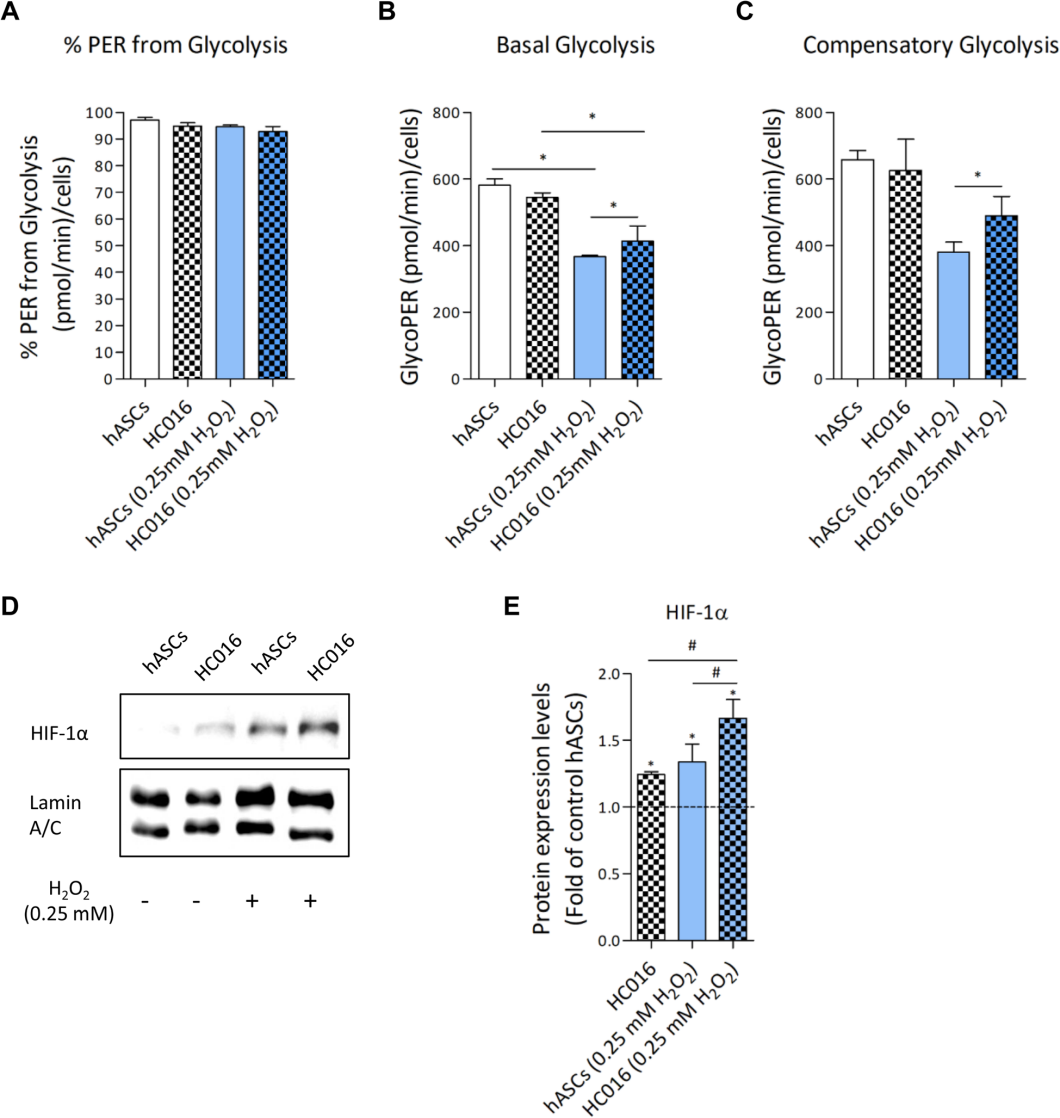
Parameters of the glycolytic activity. Both HC016 cells and hASCs present a glycolytic phenotype as indicated by **a** percentage of proton efflux rate (PER, the number of protons exported by cells into the assay medium over time) from glycolysis. Preconditioning enhanced glycolytic metabolism after 0.25 mM H_2_O_2_ exposure as indicated by **b** basal glycolysis, **c** compensatory glycolysis, **d** HIF-1α expression, and **e** quantification normalized to Lamin A/C and normalized to levels in control hASCs (dotted line). At least three different experiments were performed. Data were expressed as mean ± SD. *p < 0.05, compared with control hASCs, ^#^*p* < 0.05

Cells analyzed by XF Glycolytic Rate Assay showed that both hASCs and HC016 cells are predominantly glycolytic, considering that more than 90% of the total rate of extracellular acidification comes from glycolysis (Fig. 5a). When exposed to 0.25 mM H_2_O_2_, lower glycolytic activity was detected in both cell types compared to their corresponding control cells; however, in the case of hASCs, preconditioned cells showed a more glycolytic phenotype as indicated by higher basal glycolysis (Fig. 5b) and higher compensatory glycolysis (Fig. 5c). These results were consistent with HIF-1α overexpression in HC016 cells exposed to H_2_O_2_ (Fig. 5d), the level of HIF-1α expression being 1.25-fold higher in these cells than in oxidized hASCs (Fig. 5e).

**Fig. 6:**
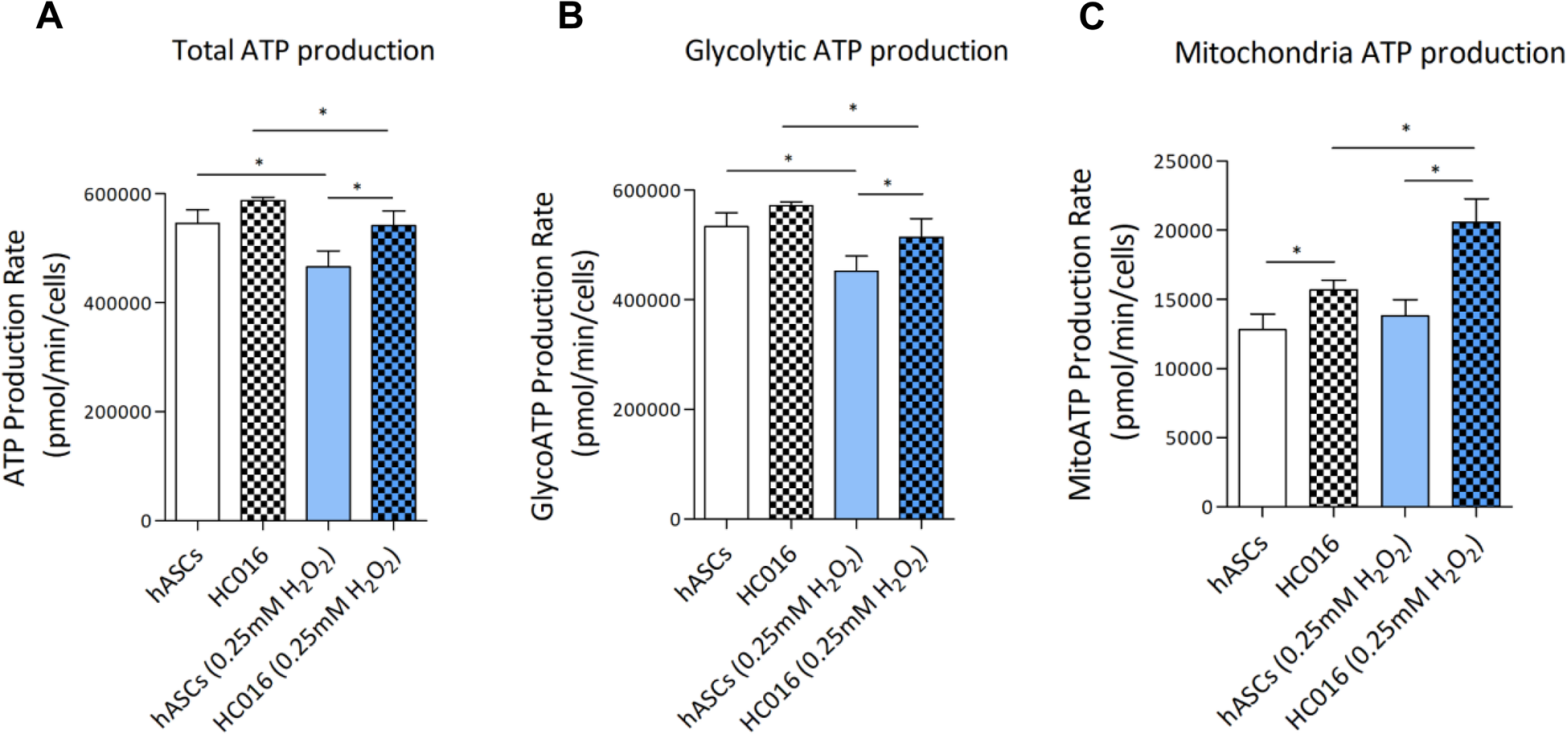
Quantification of ATP production rate. Seahorse XF Real-Time ATP Rate Assays were performed in HC016 cells and hASCs under control conditions or after 0.25 mM H_2_O_2_ exposure. **a** Total ATP production rate, **b** glycolytic ATP production rate, and **c** mitochondrial respiration ATP production rate. At least three different experiments were performed and data were expressed as mean ± SD. *p < 0.05

In addition, we analyzed the total cellular ATP production rate as well as the fractional contribution from glycolysis and oxidative phosphorylation, simultaneously. Figure 6a revealed that oxidative stress significantly decreases ATP production, this drop being more acute in hASCs (decrease of 17 ± 1%) than in HC016 cells (decrease of 9.8 ± 0.5%). The same pattern was observed in glycolytic ATP production (decrease of 17.5 ± 0.9% and 10.1 ± 1%, respectively) which, as expected, was the main source of ATP for hASCs and HC016 cells (Fig. 6b). Finally, mitochondrial ATP production (which accounted for around 5% of total ATP) was 19.2 ± 1% higher in HC016 cells than in hASCs, and this difference became more marked when the cells were exposed to moderate oxidative stress (increase of 32.3 ± 2%). Hence, whereas no significant differences were detected between control and H_2_O_2_-exposed hASCs, ATP production was 22.9 ± 2.3% higher in 0.25 mM H_2_O_2_-exposed HC016 cells than their corresponding control (Fig. 6c).

## Discussion

The therapeutic efficacy of MSCs is limited due to their low cell survival rate and, subsequently, poor engraftment at the site of injury [5]. MSC preconditioning is one of the main strategies for tackling this problem and improving their therapeutic effectiveness [27]. Since one of the main reasons why MSCs die following engraftment is ROS-mediated oxidative stress [24], several studies have already focused on the beneficial effect of preconditioning cells with sub-lethal doses of H_2_O_2_ [1–100 μM]. For instance, preconditioned human umbilical cord MSCs were found to be more resistant to oxidative damage induced by high-concentration H_2_O_2_ exposure than controls, which showed a significantly lower cell number than the preconditioned group [33]. H_2_O_2_ preconditioning also protected rat bone marrow MSCs against in vitro apoptosis [34]. In line with this, it has been described that, in human Wharton jelly MSCs, preconditioning increases survival after exposure to toxic levels of H_2_O_2_ by overexpression of HIF-1α protein [35].

In addition, we have previously reported that H_2_O_2_ preconditioning was associated with hASCs having a significantly faster recovery capacity post-thaw and an enhanced capacity to respond to oxidative stress and nutrient deprivation [31]. Further, these preconditioned cells have shown neuroprotective effects in a rat model of acute spinal cord injury, with a greater survival capacity in injured areas than control cells [36]. Nonetheless, the exact mechanisms underlying the MSC preconditioning process remain to be fully understood. In the current study, we evaluated the preconditioning effect of low doses (10 μM) of H_2_O_2_ on hASC behavior, thereby elucidating bioenergetic and molecular mechanisms underpinning the survival and adaptation of these cells under oxidative stress.

Firstly, we assessed the survival of preconditioned hASCs under oxidative conditions. For this, H_2_O_2_ was administered directly to the culture medium at two different concentrations (0.25 or 0.5 mM) to induce oxidative stress in the cells. The H_2_O_2_ remained in contact with the cells for 1 h, during which a progressive increase in intracellular levels of ROS confirmed that the application of oxidation by a single pulse of H_2_O_2_ was a valid method for conducting this study. H_2_O_2_ produces a time- and dose-dependent increase in ROS levels, which is probably related to cytotoxicity and apoptosis. Notably, HC016 cells tolerated different concentrations of H_2_O_2_ better than hASCs, levels of intracellular ROS, cytotoxicity, and apoptosis being lower than in non-preconditioned cells. This greater tolerance was most evident when cells were exposed to a concentration of 0.25 mM H_2_O_2_, which is similar to that observed in inflammatory processes [37, 38], and hence, from this point onwards, we only used that concentration for the remaining experiments.

The resistance of these cells to oxidative stress, and therefore their greater survival, is probably due to the activation of several different signaling pathways. Since apoptosis and intracellular ROS levels of HC016 cells exposed to 0.25 or 0.5 mM H_2_O_2_ were significantly lower than those observed in hASCs, we evaluated the Nrf2-ARE signaling pathway. Several authors have reported that the modulation of Nrf2 activity and its downstream antioxidant enzyme expression influence survival, apoptosis, and ROS production in MSCs [39–41]. MSCs constitutively express enzymes required to manage oxidative stress [42], and our results showed that this expression can be enhanced with H_2_O_2_ preconditioning. Compared to non-preconditioned hASCs, HC016 cells exhibited higher expression of Nrf2 and antioxidant and detoxification enzymes related to this transcription factor, HO-1, SOD-1, GPx-1, and CAT, upon oxidative stimulation. These results suggest that the antioxidant status of HC016 cells is likely to be responsible for the enhanced cell survival capacity observed.

Previous reports have also described that Nrf2 activity is closely linked to the NF-κB pathway under conditions of stress in several different cell lines [15]. Specifically, pre-stimulation of Nrf2 in primary peritoneal macrophages reduces the production of COX-2, TNFα, inducible nitric oxide synthase, and IL-1β in response to lipopolysaccharide [43]. Similarly, an Nrf2-mediated increase in HO-1 expression inhibited NF-κB activity in pre-stimulated PC3 cells [44], and consistent with this, mouse embryonic fibroblasts from NF-κB-p65-knock-out mice showed reduced mRNA and protein levels of Nrf2 as well as the protein levels of HO-1 [45]. These data suggest that the downregulation of the NF-κB signaling pathway observed in HC016 cells exposed to oxidative stress is attributable to the overexpression of Nrf2 following H_2_O_2_ preconditioning. Therefore, these results imply that HC016 cells might regulate H_2_O_2_-induced inflammatory responses and oxidative stress via attenuating the activation of NF-κB and promoting the expression of Nrf2 and, consequently, the transcription of antioxidant and detoxification enzymes.

To support an enhanced antioxidant response against oxidative stress, cells need to adapt their cellular metabolism. Metabolism not only provides energy for cell survival and proliferation but also plays an important role in cell signaling and adaptation to the immediate environment [20]. Although the beneficial effects of H_2_O_2_ preconditioning in MSCs have already been described, to our knowledge, this is the first report that evaluates how it affects the bioenergetic adaptation of hASCs under oxidative stress. In this study, we have explored the two main metabolic pathways, mitochondrial respiration and glycolysis, under standard conditions and oxidative stress. MSCs have a mixed metabolism utilizing both glycolysis and oxidative phosphorylation for ATP generation, though they rely mainly on the glycolytic pathway [46–48]. The results of the current study are consistent with these previous observations, around 95% of the total ATP production of these cells being obtained from glycolysis. It seems that MSCs prefer to produce energy by glycolysis to avoid the production of ROS by mitochondrial respiration [49].

When exposed to oxidative stress, ATP production decreased significantly in both HC016 cells and hASCs, this drop being more acute in hASCs (decrease of 17 ± 1%) than HC016 cells (decrease of 9.8 ± 0.5%). When analyzing the two pathways separately, we observed that after the H_2_O_2_ insult, HC016 cells exhibited a 1.8-fold increase in basal respiration and a 1.4-fold increase in maximal respiration. Although ROS are generated by various organelles, mitochondria are the main source of cellular oxidants, and therefore, the main site of the potential overproduction of ROS [50]. Under oxidative stress, hASCs seem to limit mitochondrial activity in an attempt to reduce intracellular ROS levels, whereas HC016 cells increase it, as they have a higher concentration of antioxidants that can counteract ROS. This evidence has been further supported by findings concerning the morphology of HC016 cell mitochondria, which were more mature and elongated in shape, and MMP values, which were 14% higher in HC016 cells than in hASCs, this correlating with a higher respiration rate. On the other hand, the OXPHOS metabolic pathway accounts for only around 5% of MSC metabolism, and hence, we needed to analyze glycolysis to understand the effect of preconditioning on energy metabolism.

After the oxidative insult, we observed that basal glycolysis was significantly decreased. The reduction was more notable in hASCs, which led us to investigate underlying mechanisms that might be activated to counteract oxidative stress and enhance glycolysis in HC016 cells. In relation to this, HIF-1α is known to be a key molecule that codes for proteins related to glycolytic energy metabolism, not only under hypoxia but also under normoxia [51, 52]. Recent studies have shown that HIF-1α silencing decreases cellular glycolytic capacity, independently of mitochondrial respiration [53]. Additionally, Del Rey et al. observed an increase in apoptotic markers and a significant reduction in cell viability after HIF-1α knockdown under normal oxygen conditions [54], which provides strong evidence that HIF-1α plays an important role in cell proliferation and survival processes. Moreover, some studies have pointed to the importance of aerobic glycolysis in normal proliferating cells as a mechanism for minimizing oxidative stress [55], considering that pyruvate, generated by glycolytic metabolism, may be an efficient scavenger of ROS and therefore protect cells from oxidative stress [56]. Given this and that HIF-1α can be activated by ROS under normoxia [57, 58], we analyzed HIF-1α expression under stress conditions and detected higher levels in HC016 cells than hASCs. This finding suggests that preconditioning activates HIF-1α, thereby increasing basal glycolysis upon oxidative stimuli, likely as a mechanism to reduce intracellular ROS levels. Nevertheless, further analysis will be necessary to identify the specific pathways regulated by HIF-1α under normal O_2_ conditions.

## Conclusions

This study shows that preconditioning with low doses of H_2_O_2_ enhances survival and adaptation of hASCs under oxidative stress through two mechanisms, namely, antioxidant activity and metabolic plasticity. In this way, HC016 cells reduce intracellular ROS levels and attenuate the inflammatory response resulting from oxidative stress by overexpressing antioxidant molecules. Moreover, they are able to meet the bioenergetic demand required to survive under stress thanks to the adaptation of their energy metabolism. Taken together, H_2_O_2_ preconditioning could potentially increase the therapeutic effect of hASCs, on the one hand, increasing their survival after implantation, and on the other, promoting the secretion of factors necessary to counteract oxidative stress.

## Data Availability

All data generated or analyzed supporting conclusions are included in the current manuscript.

## Abbreviations

ARE: Antioxidant response element
CAT: Catalase
COX-2: Cyclooxygenase 2
ECAR: Extracellular acidification rate
GlycoPER: Glycolytic proton efflux rate
GPX-1: Glutathione peroxidase
H_2_O_2_: Hydrogen peroxide
hASCs: Human adipose-derived stem cells
HC016: H_2_O_2_-preconditioned hASCs
HIF-1α: Hypoxia-inducible factor 1α
HO-1: Heme-oxygenase 1
MSCs: Mesenchymal stem cells
NF-κB: Nuclear factor kappa beta
NRF2: Nuclear factor E2-related factor 2
OCR: Oxygen consumption rate
OXPHOS: Oxidative phosphorylation
PER: Proton efflux rate
ROS: Reactive oxygen species
SOD-1: Superoxide dismutase
MMP: Mitochondrial membrane potential
